# From infancy to adulthood—Developmental changes in pulmonary quantitative computed tomography parameters

**DOI:** 10.1371/journal.pone.0233622

**Published:** 2020-05-29

**Authors:** Joshua Gawlitza, Frederik Trinkmann, Franziska Trudzinski, Heinrike Wilkens, Arno Bücker, Jonas Stroeder, Peter Fries

**Affiliations:** 1 Clinic of Diagnostic and Interventional Radiology, Saarland University Medical Center, Homburg, Germany; 2 1^st^ Department of Medicine (Cardiology, Angiology, Pulmonary and Intensive Care), University Medical Center Mannheim, Medical Faculty Mannheim, Heidelberg University, Mannheim, Germany; 3 5^th^ Department of Medicine, Saarland University Medical Center, Homburg, Germany; Clinic for Infectious and tropical diseases, Clinical centre of Serbia, SERBIA

## Abstract

**Purpose:**

Quantified computed tomography (qCT) is known for correlations with airflow obstruction and fibrotic changes of the lung. However, as qCT studies often focus on diseased and elderly subjects, current literature lacks physiological qCT values during body development. We evaluated chest CT examinations of a healthy cohort, reaching from infancy to adulthood, to determine physiological qCT values and changes during body development.

**Method:**

Dose-optimized chest CT examinations performed over the last 3 years using a dual-source CT were retrospectively analysed. Exclusion criteria were age >30 years and any known or newly diagnosed lung pathology. Lung volume, mean lung density, full-width-at-half-maximum and low attenuated volume (LAV) were semi-automated quantified in 151 patients. qCT values between different age groups as well as unenhanced (Group 1) and contrast-enhanced (Group 2) protocols were compared. Models for projection of age-dependant changes in qCT values were fitted.

**Results:**

Significant differences in qCT parameters were found between the age groups from 0 to 15 years (p < 0.05). All parameters except LAV merge into a plateau level above this age as shown by polynomial models (r2 between 0.85 and 0.67). In group 2, this plateau phase is shifted back around five years. Except for the volume, significant differences in all qCT values were found between group 1 and 2 (p < 0.01).

**Conclusion:**

qCT parameters underly a specific age-dependant dynamic. Except for LAV, qCT parameters reach a plateau around adolescence. Contrast-enhanced protocols seem to shift this plateau backwards.

## Introduction

Quantitative computed tomography (qCT) is an emerging diagnostic method in thoracic radiology. Already in the early stages of computed tomography, quantification of the usually visually assessed emphysema of the lung was published. [1] Since these first quantitative attempts CT technique changed rapidly, allowing lower doses at higher image quality including better spatial resolution. [2, 3] In consequence, the use of qCT became more common in several fields of research. One field of extensive research in this area is chronic obstructive pulmonary disease (COPD). In several previous works, correlations between qCT and lung function values were shown in patients with COPD. [4–7] Other studies were able to show correlations between exacerbation rate or mortality and qCT values. [8–10] Moreover, few works have focused on the prediction of lung function values from qCT values. [11, 12] On the basis of this evidence, the American Thoracic Society and the European Respiratory Society released a statement, substantiating the relevance of research in the field of qCT and COPD. [13] With further developments in dose reduction and image quality optimization (e.g. spectral shaping, iterative reconstruction), even research in young adults and children became feasible and more common. [14–17] Besides COPD, cystic fibrosis and interstitial lung disease are potential indications for the qCT. [18] Several studies have found diagnostic value of quantitative computed tomography in interstitial lung disease showing correlations between qCT findings and mortality as well as physiological values. [19, 20] Further, qCT has been used to objectify air trapping in children with cystic fibrosis as early as 1998. [21] For several other disease such as asthma but also lymphangioleiomyomatosis qCT was shown to be useful as objective diagnostical parameter. [22, 23] Crucial for the potential implementation of qCT in the diagnostical workflow is the understanding of its findings in context of age and the differentiation of qCT values in healthy and pathological lungs. Based on the current data, these “normal values” are often missing. Most studies only included ill patients of one age group in different disease stages to analyze for example correlations of qCT values and lung function testing—either in adults or children. [4, 19, 24, 25]

In response, the aim of this retrospective study was to determine lung qCT parameters in a cross-sectional, lung-healthy cohort of patients at different ages, reaching from infancy to adulthood. Thereby, potential changes and trends in qCT parameters during body development should be detected. To represent a wider spectrum, CT examinations with and without contrast agent were separately examined in this study.

## Methods

### Study design

We retrospectively analyzed thoracic CT examinations of patients, performed at our local medical university hospital center on a 3^rd^ generation dual source CT between 2016 and 2019. Exclusion criteria were age above 30 years, lung pathologies of any kind (e.g. on-going inflammation, neoplasia, asthma, reduced lung function values) in patient´s history as well as radiological lung pathologies (opacities, fibrosis, emphysema, scars). The latter was determined by an experienced radiologist (15 years of experience). If available, height and weight of patients was obtained via the DICOM metadata.

We have obtained a waiver from the local institutional review board (Institutional Review Board of the Medical Association Saarland) for this study. The Institutional Review Board of the Medical Association Saarland is simultaneously our local institutional review board. Further, we have written consent of all patients / legal guardians for analysis of the data. All identifying data was anonymized for final analysis.

### CT examinations

All CT examinations were performed on a 3^rd^ generation dual source CT (Somatom Force, Siemens Healthineers, Forchheim, Germany). Two protocols were used in the analyzed cohort. First, an ultra-low-dose, non-contrast enhanced chest CT (Group 1) with scan parameters as follows: 100 kVp tube voltage with spectral shaping using a dedicated tin filter technique, automated tube current modulation using 96mAs as reference, 0.25 s rotation time, pitch 1.2, 192 mm × 0.6 mm detector collimation. All images were reconstructed with a slice thickness of 1 mm, using a suitable reconstruction kernel for quantitative lung analysis (Br40) and a 3^rd^ generation iterative reconstruction technique (Adaptive Model-based Iterative reconstruction [ADMIRE], Siemens Healthineers, Germany). The reconstruction algorithm was substantially explained in a previous publication by Gordic et al. [26] An iterative strength level of three was chosen for the present study for optimum image noise as recommended by the CT vendor for quantitative lung analysis.

Second, a low dose-chest contrast enhanced CT was evaluated (Group 2). Scan parameters were as follows: tube voltage between 70 and 100 kVp depending on automated tube voltage regulation and age. Reference tube current varied correspondingly between 172 and 933 mAs. The pitch varied from 0.8 to 2.8, where younger patients received predominantly high pitch examinations. Highly concentrated iodinated contrast medium (Imeron 400, Bracco Imaging S.p.A., Italy) was applied in all patient through peripheral venous access catheters. In total 5 to 70 ml of contrast media, depending on the body weight, were injected at a flow rate of 1 to 3 ml/s, followed by a saline chaser (20 ml) at the same flow rate using a power injector (Accutron CT-D, Medtron AG, Saarbrücken, Germany). Reconstruction and postprocessing techniques were identical to the prior protocol.

### Image analysis

**Fig 1:**
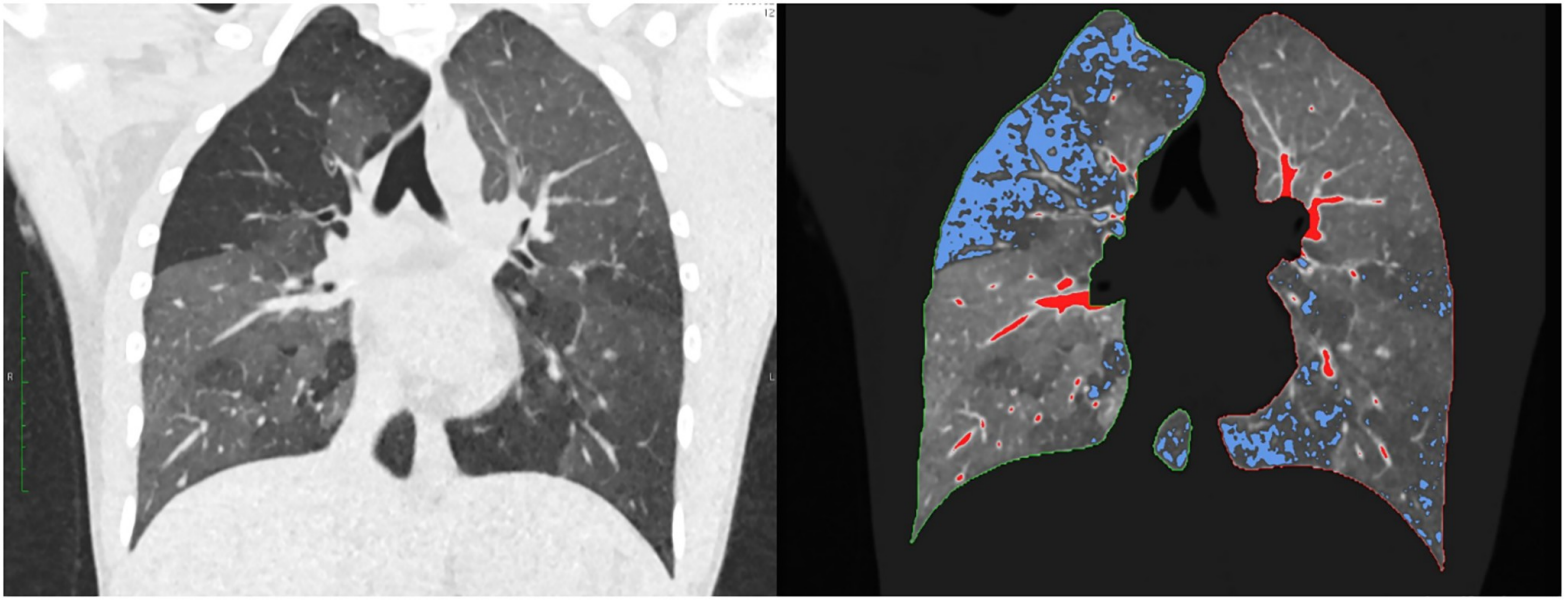
Lung quantification. Coronary reformation; automatic detection of lung borders and lung parenchyma. blue areas: low attenuation volume (LAV) with HU values below -950, red areas: high attenuation volume (HAV) with HU values above -200. This patient was not included in the analysis but showcases the quantitative analysis in low attenuated areas.

Datasets were analyzed using dedicated semi-automatic software (SyngoViaVB30, Pulmo3D, Siemens Healthineers, Forchheim, Germany). Lung segmentation was automated and manually revised if necessary (Fig 1). Four quantitative parameters were acquired: total lung volume (volume), mean lung density (MLD), full-width-half-max (FWHM) and low attenuation volume (LAV). The LAV threshold for emphysema was set to -950 HU. This cut-off had been extensively evaluated in previous studies and strongly correlates with microscopic and gross emphysema [27, 28]. FWHM marks the width at the half maximum of the voxel count to specific HU value curve representing the density distribution of the lung parenchyma. A graphical explanation of the latter can be found in the online supplement. (S1 Fig).

### Statistical analysis

All statistics have been performed, using a dedicated statistic program (JMP 13, Cary, USA). Mean, median and standard deviation have been calculated for all parameters, with regard to the protocols used. In a second step, the patients of the two groups (contrast enhanced, non-contrast enhanced) were summarized in order of their age. Patients were clustered in 6 subgroups according to a five-year range of age (0–5 years, 6–10 years, 11–15 years, 16–20 years, 21–25 years, 26–30 years). The quantitative parameters were then compared between these groups to find age-related changes using ANOVA corrected for multiple testing using Tukey HSD. Further, to specify these changes in specific parameters over age, regression models were calculated, using R Statistical Software (v3.4.2, Foundation for Statistical Computing, Vienna, Austria). [29, 30]

## Results

### Patient collective

Initially 471 chest CT examinations performed in patients under 30 years at the given timeframe were found. After applying the exclusion criteria, 320 cases had to be rejected from the study cohort, leaving 151 cases for final analysis– 53 patients in group 1, 98 in group 2. Group 1 consisted of 25 females and 28 males, group 2 of 35 females and 63 males (p-value Chi square = 0.223). The mean age of group 1 was 15 years with a standard deviation of 7 years. The youngest patient was 2 years, the oldest 30 years old. The mean age in group 2 was 19 years with a standard deviation of 8 years. The youngest patient was 2 months old, the oldest being 30 years. The specific age distribution over the groups and age-related subgroups can be found in Table 1.

**Table 1:** Specific age distribution over groups and subgroups.

subgroup	group	n	mean age	minimum	maximum	SD
1–5 years	Group 1	8	3.3	2	5	1.5
	Group 2	10	2	0	5	1.4
6–10 years	Group 1	4	9.3	9	10	0.5
	Group 2	7	7.4	6	10	1.5
11–15 years	Group 1	14	13.1	11	15	1.5
	Group 2	11	13.5	11	15	1
16–20 years	Group 1	13	17.2	16	19	1.1
	Group 2	16	18.6	16	20	1.2
21–25 years	Group 1	8	22.6	21	25	1.2
	Group 2	24	23.4	21	25	1.4
26–30 years	Group 1	6	27.7	26	30	1.6
	Group 2	30	27.6	26	30	1.4

Shown is the age distribution for both CT protocols examined (“group”) as well as the age-related subgroups (“subgroup”).

No statistical differences were found regarding mean body size (154 ± 30 cm Group 1 vs. 161 ± 35 cm Group 2; p-value = 0.414) and mean body weight (52 ± 26 kg Group 1 vs 66 ± 33 kg Group 2; p-value = 0.1) between both groups.

Regarding the clinical indication for the examinations, 68 patients (45%) received the CT due to suspected pulmonary metastasis. 48 patients (32%) received the examinations due to suspected vascular anomalies (e.g. double aortic arch) and in 24 cases (16%) a pneumonia was suspected. The remaining examinations were performed due to other clinical indications (e.g. suspected bone anomalies).

### Quantitative CT values

Results of qCT values are given as means ± standard deviation. Mean total lung volume in group 1 was 3.3 ± 1.7 l. The lowest volume measured was 0.34, the highest 7 l. The corresponding volumes for group 2 were 3.4 ± 1.8 l with a minimum of 0.15 and a maximum of 7.7 l, respectively. No significant difference was found between both groups (p-value = 0.65, Student’s t-test).

MLD in group 1 was -778 ± 89 HU with the lowest measured MLD of -872 HU and the highest at -500 HU. In group 2 the mean MLD was -713 ± 112 HU, the lowest -847 HU and the highest -383 HU. The MLD varied significantly between both groups (p-value = 0.0001).

Mean FWHM in group 1 was 109 ± 46 HU with the highest at 246 HU and the lowest at 54 HU. The mean FWHM in group 2 was 132 ± 61 HU with the highest at 373 HU and the lowest at 68 HU. Significant differences were found for FWHM between group 1 and 2 (p-value = 0.01).

**Fig 2:**
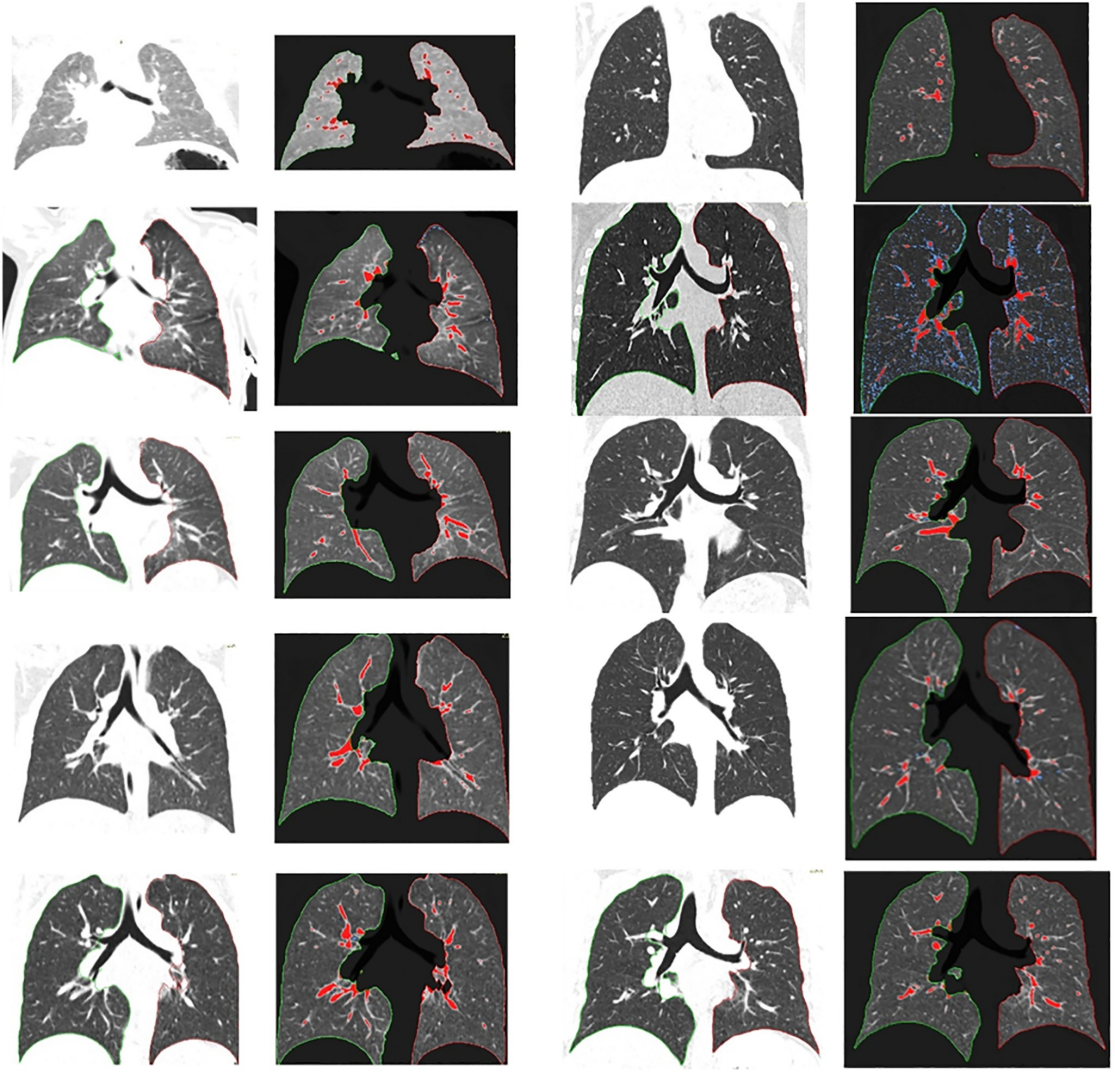
Parenchyma quantification from infancy to adulthood. Reading direction: left top to bottom, right top to bottom. Age as following: 0, 3, 6, 9, 11, 15, 18, 21, 24, 27 years. Shown are exemplary lung parenchymas with (right images) and without (left images) quantification. Red dyed parenchyma shows HU values above -200, blue tissue below -950.

Mean LAV in group 1 was 1.2 ± 1.5%. The highest measured percentage was 8.1%, the lowest 0%. In group 2, the mean LAV was 0.5 ± 1.2% with a maximum of 10% and a minimum of 0% as well. The quantitative percentage between both groups showed significant differences (t-value = 2.6, p-value = 0.01) with details given in Table 2. Exemplary images of quantitative lung parenchyma for every subgroup can be found in Fig 2.

**Table 2:** Mean values and t-test for comparison between groups.

	Volume	MLD	FWHM	LAV
Group 1	3.2 ± 1.8	-778± 89	109 ± 46	1.2 ± 1.5
Group 2	3.3 ± 1.7	-713 ± 112	132 ± 61	0.5 ± 1.2
p-value	0.32	0.0001	<0.01	0.01

Shown are the mean values with standard deviation of the four quantitative lung parameters for both protocols used.

Volume is shown in liter, mean lung density (MLD) in HU, full width at half maximum (FWHM) in HU and low attenuated volume in percentage of total lung volume. Further, the p-value shows the significance level of the t-test.

### Age dependency of parameters

**Fig 3:**
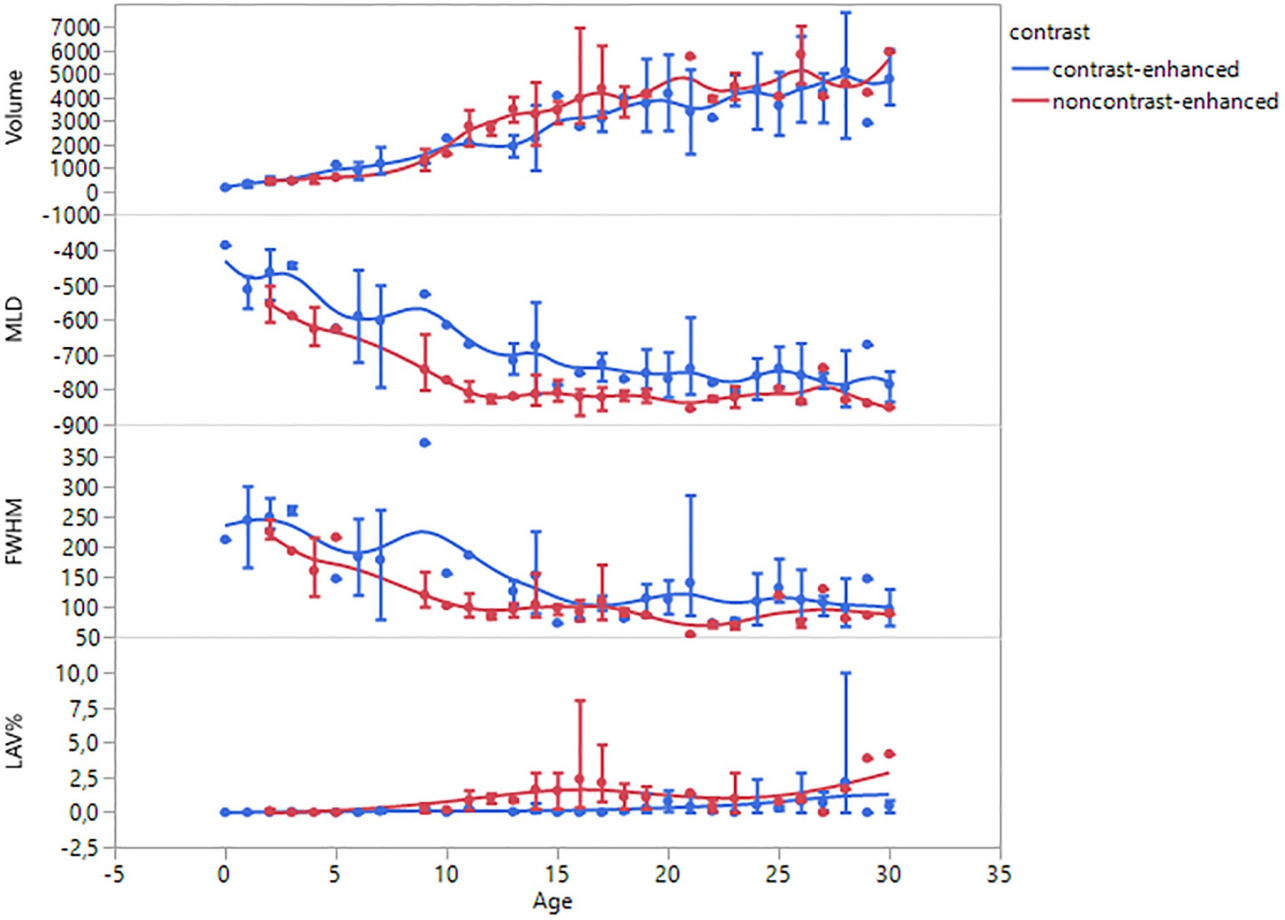
qCT parameters against age. Shown are the mean changes in the four measured parameters of the age of the patients with standard deviation and smoothed trend lines. As shown, while volume and emphysema percentage (LAV) rise over time, mean lung density (MLD) and the full width at half maximum of the volume density histogram (FWHM) decline.

As represented by Fig 3, all measured parameters showed an age-dependant dynamic development. The mean values with standard deviation for the six age subgroups can be found in Table 3. As shown in the ANOVA results, there are significant differences between the age groups for every parameter except the LAV in group 1 (p = 0.05).

**Table 3:** Mean values of quantitative lung parameters for different age groups.

		0–5	6–10	11–15	16–20	21–25	26–30	p-value
**Volume**	Group 2	0.4±0.2	1.3±0.6	2.3±1	3.7±1	3.9±1	4.6±1.3	<.0001
	Group 1	0.5±0.5	1.4±0.4	3.2±0.8	4±1.3	4.4±0.6	5.1±1.2	<.0001
**MLD**	Group 2	-480±76	-587±126	-697±81	-750±43	-755±51	-772±56	<.0001
	Group 1	-592±55	-748±74	-812±27	-818±25	-823±23	-819±42	<.0001
**FWHM**	Group 2	236±13	204±16	138±12	109±11	115±9	105±8	<.0001
	Group 1	196±42	116±29	98±21	97±24	75±19	89±21	<.0001
**LAV**	Group 2	0.01±0.03	0.1±0.2	0.1±0.2	0.3±0.5	0.4±0.6	1.2±2	0.0173
	Group 1	0.04±0.1	0.3±0.3	1.3±0.9	1.8±2.3	0.8±1	2±1.7	0.0504

Shown are the mean values with standard deviation of the four quantitative lung parameters for every age group. Volume is shown in liter, mean lung density (MLD) in HU, full width at half maximum (FWHM) in HU and low attenuated volume in percentage of total lung volume.

The p-value shows the significance level of the ANOVA, suggesting significant difference between age groups for every parameter, except the LAV in group 1.

As shown in post-hoc analysis, especially the differences between the youngest and oldest patients were significantly (e.g. MLD_0-5_ vs MLD_26-30_: difference = 292.4 HU; p-value = <0.0001). This trend can be found for all parameters, except the LAV. No significant differences in LAV were found in the post-hoc analysis between the age subgroups.

As illustrated in Fig 3, most parameters reach a plateau phase at some age. This visual observation is underlined by the Tukey HSD. For the total lung volume, no differences were found between the age of 16–30 in both protocols.

For the MLD there were different results, regarding group 1 and 2. For group 1, no significant differences in MLD were found between the age of 11–30. For group 2, the significant differences between the MLD_11-15_ and MLD_26-30_ (p = 0.02) were shown. No significant differences were found for the subgroups above the age of 16, suggesting a homogeneous MLD as recent as the age range of 16–30.

Similar discrepancies can be found for the FWHM. In group 1, the FWHM showed no significant differences between the age subgroups from 6 to 30 years. Whereas in group 2, significant differences between FWHM_6-10_ and all older subgroups were shown (p = < 0.0328), suggesting the plateau level for FWHM between 11–30 years.

All post-hoc analysis can be found in the online supplement. (S1–S8 Tables).

Beyond the comparison of age groups, a sex-dependant analysis of quantitative parameters was performed for every age group. Statistical differences between male and female subjects were only found between the age of 16 and 25 for volume, MLD and LAV. No statistical differences for FHWM were found at any age group between the genders. (S9 and S10 Tables).

### Regression models

**Fig 4:**
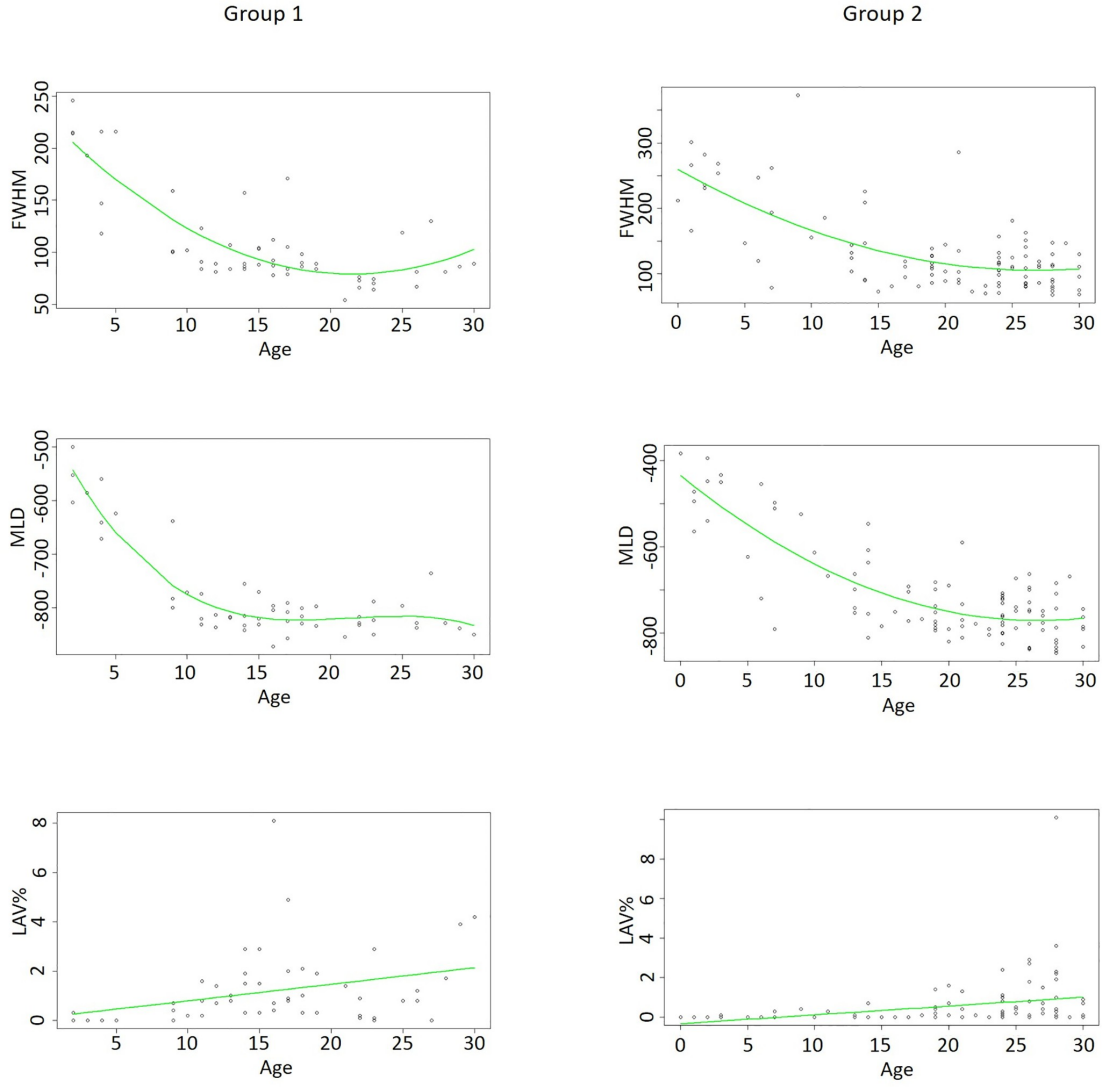
Regression models. Shown are the regression models for quantitative CT parameters—left row for group 1, right row group 2. For MLD and FWHM polynomial regression was used. In LAV linear regression models. For MLD a R-squared of 0.85 (group 1) and 0.67 (group 2) were calculated. For FWHM R-squared was 0.68 (group 1) and 0.48 (group 2), respectively. For LAV R-squared of 0.1 (both groups) were calculated.

For both groups, regression models were calculated to predict the development of all parameters. (compare Fig 4).

For the model fit of MLD a non-linear model seemed most suitable after initial, poor performance of standard linear models (R^2^ = < 0.02). In group 1, a polynomial regression was used to describe the data (R^2^ = 0.85). In group 2, 2^nd^ degree polynomial regression already described the dataset similar to 3^rd^ degree regression (R^2^ = 0.677 vs. 0.678).

Similar to the MLD, FWHM showed a non-linear relation. (compare Fig 4) For both groups, a 2^nd^ degree polynomial regression showed best data description with an R^2^ of 0.68 for group 1 and 0.48 for group 2.

In contrast to MLD and FWHM, LAV showed a linear progression over the age range. Thereby, we fit a linear regression with an R^2^ of 0.11 in group 1 and 0.1 in group 2, respectively.

## Discussion

The aim of this study was to determine mean qCT values of healthy lung tissue and their changes during body development from infancy to adulthood.

As shown, all parameters except the total lung volume showed significant differences, when comparing analysis of non-contrast enhanced (group 1) with contrast enhanced CT datasets (group 2). This is an important finding of our study, as known data primarily originates from non-contrast enhanced scans of the lung. Obviously, application of contrast media has an impact on the mainly attenuation-based analysis of qCT lung parameters. As a consequence, these parameters cannot be transferred from one examination to another and should only be compared with similar protocols.

Nonetheless, all four measured parameters underly a clear trend during aging regardless of the scan technique applied. (Fig 3).

Volume and MLD for example, underly a contrary evolution over development. As a result of increased volume and ventilation of the lung during aging, the MLD is constantly decreasing. As shown, the MLD underlies a non-linear time course and can be described by polynomial regression. The mean density reaches an even level at around 11–15 years in the non-contrast enhanced protocol group (group 1) and at 16–20 in the contrast enhanced protocol group (group 2). Similar results, suggesting a plateau in MLD around this age, were found by Gevenois et al. They did not find significant differences of MLD in individuals between 21 and 70. [27] The relatively high MLDs, measured for the individuals between 0 and 5 years in group 1, were confirmed by the findings of Long et al. [31] Further, Stein et al. showed a similar relationship of MLD and LAV for children between 0 and 7 years. [32]

Similar to MLD, an offset was shown for the FWHM distribution over the age range. While no significant differences were found in group 1 between age 6 and 30 (p = >0.1332), in group 2 the even level was not reached until the age group of 11–15 years. Likewise, the non-linear connection could be described by a polynomial regression.

Our results suggest, the level of equality in the quantification of contrast enhanced protocols for MLD and FWHM might be shifted a few years back. One reason for this might be the ongoing development of the lung at that age. The described offset between group 1 and 2 is during childhood and adolescence, respectively. As shown in previous studies, the number of alveoli at this age is already set and not increasing anymore. [33] Growth and expansion of the lung characterizes the ongoing body development. Thereby, especially in younger children less air per volume is inside the lung tissue. A contrast media induced increase in density might thereby be more dominant in younger lungs: as per given volume, the enhancing, parenchymatous fraction is larger than the non-enhancing air space at this age. As the airspace expands during aging, the air will make a larger portion at a given volume and thereby countervail the enhancing parenchyma.

In contrast to MLD and FWHM, no significant differences were shown for LAV over the age groups in post-hoc analysis. As LAV defines emphysema, it is not expected to be predominant in young, healthy lungs. [27] The small percentage of emphysema found might be due to abnormal parenchymal delopment as no noxae induced emphysema is expected at that age. Irion et al. found similar percentages in LAV for healthy adults between 21 and 40 years. [34] Even though no significant differences were found between the age subgroups, mean LAV of group 1 and 2 differed significantly. Similar to MLD and FWHM this is most likely due to an overall shift in density. As LAV is defined by areas with a density below -950 HU, an increasing density is leading to a reduced LAV. Thereby, lung quantification in respect to emphysema assessment is usually not recommended for contrast enhanced protocols. [35] Nonetheless, group 2, receiving such a protocol, was analysed in this study. The reason for this is the cohort itself: infants to adults. The highest priority in a young cohort is radiation protection. [36, 37] Therefore, infants, children and young adults often receive only single-phase lung CT—either with or without contrast media. Even if an additional lung quantification is desired, the protocols in children should be chosen solely by the clinical indication. If this indication requires only a contrast enhanced protocol, no additional non-contrast enhanced scan should be performed to comply with emphysema quantification criteria. Nevertheless, qCT parameters might implicate important information—even in contrast enhanced protocols. The data acquired in this work, might help future studies to categorize their measured qCT findings, regardless of applied contrast media.

Even though LAV is significantly different between group 1 and 2, both groups show a similar slow increase. As suggested by the linear regression models, LAV is rising between 0.04 and 0.07% every year—resulting at a LAV% of around 1 at the age of 20. Cho et al. found a LAV% of around 2–4.6% at the age of 50 in healthy adult non-smokers, which underlines this steady increase. [38]

As shown, all quantitative lung parameters underly a specific dynamic in the context of aging. Three of the four parameters measured reach a plateau level around the adolescence age—varying with regard to contrast media application. Previous studies have described qCT parameters in healthy, young patients before. [31, 34, 39] One drawback of the current literature is the age range. Johnson et al. as well as Long et al. for example focused only on healthy children below the age of five, Stein et al. below the age of seven. [31, 32, 39] On the other side, Irion et al., Zach et al. and Gevenois et al. for example focused only on healthy adults above 20 years. [27, 34, 40] The intention of this current study was to project the qCT parameter dynamic over a wider age range. As additionally suggested by our results, the important levelling in qCT parameters occurs right between these mentioned age ranges of 5 and 20 years.

Nonetheless, this study underlies several limitations. First, the sample size of both protocols is limited. A larger cohort size would be preferable to receive more robust mean values as well as smaller standard deviations. Especially in the comparison between the older subgroups (age 21 to 30) as well as female and male comparison more subtle changes are most likely to be missed due do small sample size. Nevertheless, especially in young children acquisition numbers of lung-healthy individuals are often limited.

A second limitation of the study is the retrospective design and its inherent projection of group findings on individuals. To get more valid data, on how qCT findings are changing during development, a prospective study with a CT scan of every individual every year would be way more preferable. Nonetheless, from an ethical and radiation protection point of view this would be unacceptable. In a similar matter, a uniform scan protocol in Group 2 would be preferable, as different kV/mAs settings might affect quantitative parameters. But as especially young children do profit from low-kV examinations, a uniform protocol would be unacceptable.

Beyond the scan protocol, due to the retrospective study design, clinical information (height, weight) could not be obtained for every patient. A prospective analysis would provide a more consistent database regarding these demographics.

Another limitation is the absence of spirometric triggering during the CT scans. Although all patients received verbal breathing commands, especially the young patients were most likely not to follow them. This variable state of breath might have influenced quantitative parameters due to variable lung density.

As shown in this study, quantitative lung function parameters underly a specific dynamic during the body development from infancy to adulthood. Our data suggests, all parameters, except the emphysema percentage, reach a constant level during adolescence. Further, contrast media application seems to shift this point of constant evenness a few years back, most likely due to overall increased lung density. Further research should be undertaken, to describe these findings in more detail and over a wider spectrum of protocols and diseases. The findings in this study might assist future research as a point of reference in regard to lung quantification of younger patients and the discrimination of healthy and pathological qCT values.

## Supporting information

S1 Fig Full width at half maximum (FWHM) measured in the voxel-density-histogram. Shown is a voxel-density histogram from a qCT. The number of voxels is represented on the y-axis while their HU values are on the x-axis. The full-width-half-max (FWHM) is the full width at the half maximum of this histogram.
(DOCX)

S1 Table Comparison of age groups regarding total lung volume—group 2 (contrast enhanced). (DOCX)

S2 Table Comparison of age groups regarding MLD—group 2 (contrast-enhanced). (DOCX)

S3 Table Comparison of age groups regarding FWHM—group 2 (contrast-enhanced). (DOCX)

S4 Table Comparison of age groups regarding LAV—group 2 (contrast-enhanced). (DOCX)

S5 Table Comparison of age groups regarding total lung volume—group 1 (non-contrast-enhanced). (DOCX)

S6 Table Comparison of age groups regarding—MLD group 1 (non-contrast-enhanced). (DOCX)

S7 Table Comparison of age groups regarding FWHM—group 1 (non-contrast-enhanced). (DOCX)

S8 Table Comparison of age groups regarding LAV—group 1 (non-contrast-enhanced). (DOCX)

S9 Table Sex-dependant comparison of age groups—group 1 (non-contrast-enhanced). (DOCX)

S10 Table Sex-dependant comparison of age groups—group 2 (contrast-enhanced). (DOCX)

## Abbreviations

COPD: chronic obstructive pulmonary disease
FWHM: full width at half maximum
LAV: low attenuated volume
MLD: mean lung density
qCT: quantitative computed tomography
