# Supplementary material for: From infancy to adulthood—Developmental changes in pulmonary quantitative computed tomography parameters
Source: PLoS One. 2020 May 29;15(5):e0233622. doi: 10.1371/journal.pone.0233622 (PMC7259551; doi:10.1371/journal.pone.0233622)
Supplement: S1 Fig — Shown is a voxel-density histogram from a qCT. The number of voxels is represented on the y-axis while their HU values are on the x-axis. The full-width-half-max (FWHM) is the full width at the half maximum of this histogram. (DOCX) [file pone.0233622.s001.docx]

| Figure S1: Full width at half maximum (FWHM) measured in the voxel-density-histogram |
| --- |
|  |
| 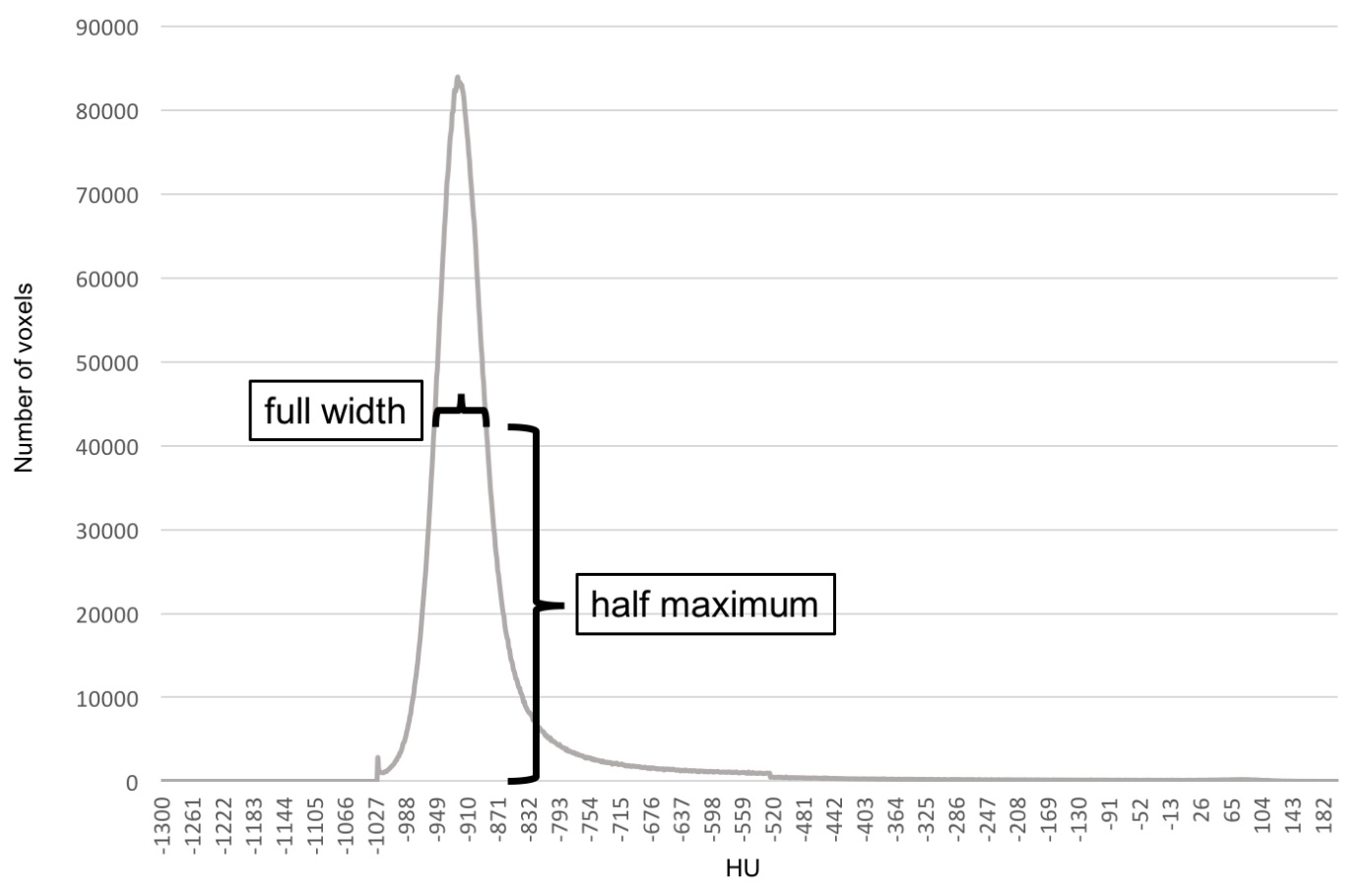 |
| Figure S1: voxel-density histogram from a qCT. The number of voxels is represented on the y-axis while their HU values are on the x-axis. The full-width-half-max (FWHM) is the full width at the half maximum of this histogram. |
