# Supplementary material for: From infancy to adulthood—Developmental changes in pulmonary quantitative computed tomography parameters
Source: PLoS One. 2020 May 29;15(5):e0233622. doi: 10.1371/journal.pone.0233622 (PMC7259551; doi:10.1371/journal.pone.0233622)
Supplement: S1 Table — (DOCX) [file pone.0233622.s002.docx]

| Table S1: comparison of age groups regarding total lung volume - Group 2 (contrast enhanced) | | | | | | |
| --- | --- | --- | --- | --- | --- | --- |
|  | | | | | | |
| **Compared groups** | | **difference** | **SE** | **Lower CI** | **Upper CI** | **p-value** |
| 0-5 | 26-30 | 4232,900 | 413,4799 | 3029,40 | 5436,399 | <,0001* |
| 0-5 | 21-25 | 3527,908 | 426,2053 | 2287,37 | 4768,447 | <,0001* |
| 0-5 | 16-20 | 3223,450 | 456,4694 | 1894,82 | 4552,077 | <,0001* |
| 0-5 | 11-15 | 1838,291 | 494,7643 | 398,20 | 3278,382 | 0,0046* |
| 0-5 | 6-10 | 814,486 | 558,0337 | -809,76 | 2438,732 | 0,6905 |
| 11-15 | 26-30 | 2394,609 | 399,1352 | 1232,86 | 3556,356 | <,0001* |
| 11-15 | 21-25 | 1689,617 | 412,3036 | 489,54 | 2889,693 | 0,0012* |
| 11-15 | 16-20 | 1385,159 | 443,5173 | 94,23 | 2676,087 | 0,0280* |
| 16-20 | 26-30 | 1009,450 | 350,5446 | -10,87 | 2029,766 | 0,0542 |
| 16-20 | 21-25 | 304,458 | 365,4680 | -759,29 | 1368,212 | 0,9606 |
| 21-25 | 26-30 | 704,992 | 310,1099 | -197,63 | 1607,616 | 0,2156 |
| 6-10 | 26-30 | 3418,414 | 475,3092 | 2034,95 | 4801,878 | <,0001* |
| 6-10 | 21-25 | 2713,423 | 486,4198 | 1297,62 | 4129,225 | <,0001* |
| 6-10 | 16-20 | 2408,964 | 513,1448 | 915,37 | 3902,554 | 0,0001* |
| 6-10 | 11-15 | 1023,805 | 547,4896 | -569,75 | 2617,362 | 0,4271 |
| Shown is the post-hoc analysis with Tukey HSD for group comparison with significance level. The first two rows show the compared groups pairs. **SE**: standard error; **CI**: confidence interval | | | | | | |
