# Supplementary material for: From infancy to adulthood—Developmental changes in pulmonary quantitative computed tomography parameters
Source: PLoS One. 2020 May 29;15(5):e0233622. doi: 10.1371/journal.pone.0233622 (PMC7259551; doi:10.1371/journal.pone.0233622)
Supplement: S2 Table — (DOCX) [file pone.0233622.s003.docx]

| Table S2: comparison of age groups regarding MLD – Group 2 (contrast-enhanced) | | | | | | |
| --- | --- | --- | --- | --- | --- | --- |
|  | | | | | | |
| **Compared groups** | | **difference** | **SE** | **Lower CI** | **Upper CI** | **p-value** |
| 0-5 | 26-30 | 292,3667 | 23,94632 | 222,667 | 362,0663 | <,0001* |
| 0-5 | 21-25 | 274,9833 | 24,68330 | 203,139 | 346,8280 | <,0001* |
| 0-5 | 16-20 | 270,0250 | 26,43602 | 193,079 | 346,9713 | <,0001* |
| 0-5 | 11-15 | 217,4455 | 28,65383 | 134,044 | 300,8470 | <,0001* |
| 0-5 | 6-10 | 107,1857 | 32,31803 | 13,119 | 201,2525 | 0,0160* |
| 11-15 | 26-30 | 74,9212 | 23,11556 | 7,640 | 142,2027 | 0,0200* |
| 11-15 | 21-25 | 57,5379 | 23,87819 | -11,963 | 127,0392 | 0,1637 |
| 11-15 | 16-20 | 52,5795 | 25,68591 | -22,183 | 127,3425 | 0,3244 |
| 16-20 | 26-30 | 22,3417 | 20,30148 | -36,749 | 81,4324 | 0,8800 |
| 16-20 | 21-25 | 4,9583 | 21,16576 | -56,648 | 66,5647 | 0,9999 |
| 21-25 | 26-30 | 17,3833 | 17,95974 | -34,891 | 69,6580 | 0,9269 |
| 6-10 | 26-30 | 185,1810 | 27,52711 | 105,059 | 265,3030 | <,0001* |
| 6-10 | 21-25 | 167,7976 | 28,17057 | 85,803 | 249,7926 | <,0001* |
| 6-10 | 16-20 | 162,8393 | 29,71832 | 76,339 | 249,3392 | <,0001* |
| 6-10 | 11-15 | 110,2597 | 31,70738 | 17,970 | 202,5491 | 0,0098* |
| Shown is the post-hoc analysis with Tukey HSD for group comparison with significance level. The first two rows show the compared groups pairs. **MLD**: mean lung density; **SE**: standard error; **CI**: confidence interval | | | | | | |
