# Supplementary material for: From infancy to adulthood—Developmental changes in pulmonary quantitative computed tomography parameters
Source: PLoS One. 2020 May 29;15(5):e0233622. doi: 10.1371/journal.pone.0233622 (PMC7259551; doi:10.1371/journal.pone.0233622)
Supplement: S3 Table — (DOCX) [file pone.0233622.s004.docx]

| Table S3: comparison of age groups regarding FWHM – Group 2 (contrast-enhanced) | | | | | | |
| --- | --- | --- | --- | --- | --- | --- |
|  | | | | | | |
| **Compared groups** | | **difference** | **SE** | **Lower CI** | **Upper CI** | **p-value** |
| 0-5 | 26-30 | 131,0000 | 16,17661 | 83,9154 | 178,0846 | <,0001* |
| 0-5 | 16-20 | 127,6125 | 17,85849 | 75,6325 | 179,5925 | <,0001* |
| 0-5 | 21-25 | 121,1333 | 16,67447 | 72,5996 | 169,6670 | <,0001* |
| 0-5 | 11-15 | 97,5727 | 19,35671 | 41,2319 | 153,9135 | <,0001* |
| 0-5 | 6-10 | 31,8714 | 21,83200 | -31,6741 | 95,4170 | 0,6904 |
| 11-15 | 26-30 | 33,4273 | 15,61540 | -12,0239 | 78,8784 | 0,2761 |
| 11-15 | 16-20 | 30,0398 | 17,35177 | -20,4653 | 80,5449 | 0,5150 |
| 11-15 | 21-25 | 23,5606 | 16,13059 | -23,3901 | 70,5113 | 0,6899 |
| 16-20 | 26-30 | 3,3875 | 13,71439 | -36,5304 | 43,3054 | 0,9999 |
| 21-25 | 26-30 | 9,8667 | 12,13246 | -25,4468 | 45,1801 | 0,9644 |
| 21-25 | 16-20 | 6,4792 | 14,29824 | -35,1381 | 48,0965 | 0,9975 |
| 6-10 | 26-30 | 99,1286 | 18,59556 | 45,0032 | 153,2539 | <,0001* |
| 6-10 | 16-20 | 95,7411 | 20,07581 | 37,3072 | 154,1749 | <,0001* |
| 6-10 | 21-25 | 89,2619 | 19,03024 | 33,8713 | 144,6525 | 0,0001* |
| 6-10 | 11-15 | 65,7013 | 21,41949 | 3,3565 | 128,0461 | 0,0328* |
| Shown is the post-hoc analysis with Tukey HSD for group comparison with significance level. The first two rows show the compared groups pairs. **FWHM**: full width at half maximum; **SE**: standard error; **CI**: confidence interval | | | | | | |
