# Supplementary material for: From infancy to adulthood—Developmental changes in pulmonary quantitative computed tomography parameters
Source: PLoS One. 2020 May 29;15(5):e0233622. doi: 10.1371/journal.pone.0233622 (PMC7259551; doi:10.1371/journal.pone.0233622)
Supplement: S4 Table — (DOCX) [file pone.0233622.s005.docx]

| Table S4: comparison of age groups regarding LAV – Group 2 (contrast-enhanced) | | | | | | |
| --- | --- | --- | --- | --- | --- | --- |
|  | | | | | | |
| **Compared groups** | | **difference** | **SE** | **Lower CI** | **Upper CI** | **p-value** |
| 0-5 | 6-10 | 0,090000 | 0,5730255 | -1,57788 | 1,757883 | 1,0000 |
| 0-5 | 11-15 | 0,099091 | 0,5080563 | -1,37969 | 1,577870 | 1,0000 |
| 0-5 | 16-20 | 0,302500 | 0,4687327 | -1,06182 | 1,666822 | 0,9872 |
| 0-5 | 21-25 | 0,402500 | 0,4376555 | -0,87137 | 1,676366 | 0,9405 |
| 0-5 | 26-30 | 1,170000 | 0,4245882 | -0,06583 | 2,405832 | 0,0742 |
| 11-15 | 16-20 | 0,203409 | 0,4554326 | -1,12220 | 1,529019 | 0,9977 |
| 11-15 | 21-25 | 0,303409 | 0,4233803 | -0,92891 | 1,535725 | 0,9795 |
| 11-15 | 26-30 | 1,070909 | 0,4098581 | -0,12205 | 2,263867 | 0,1045 |
| 16-20 | 21-25 | 0,100000 | 0,3752865 | -0,99233 | 1,192331 | 0,9998 |
| 16-20 | 26-30 | 0,867500 | 0,3599622 | -0,18023 | 1,915228 | 0,1636 |
| 21-25 | 26-30 | 0,767500 | 0,3184411 | -0,15937 | 1,694374 | 0,1636 |
| 6-10 | 11-15 | 0,009091 | 0,5621982 | -1,62728 | 1,645459 | 1,0000 |
| 6-10 | 16-20 | 0,212500 | 0,5269306 | -1,32122 | 1,746216 | 0,9986 |
| 6-10 | 21-25 | 0,312500 | 0,4994876 | -1,14134 | 1,766339 | 0,9888 |
| 6-10 | 26-30 | 1,080000 | 0,4880786 | -0,34063 | 2,500631 | 0,2420 |
| Shown is the post-hoc analysis with Tukey HSD for group comparison with significance level. The first two rows show the compared groups pairs. **LAV**: low attenuated volume; **SE**: standard error; **CI**: confidence interval | | | | | | |
