# Supplementary material for: From infancy to adulthood—Developmental changes in pulmonary quantitative computed tomography parameters
Source: PLoS One. 2020 May 29;15(5):e0233622. doi: 10.1371/journal.pone.0233622 (PMC7259551; doi:10.1371/journal.pone.0233622)
Supplement: S5 Table — (DOCX) [file pone.0233622.s006.docx]

| Table S5: comparison of age groups regarding total lung volume - Group 1 (non-contrast-enhanced) | | | | | | |
| --- | --- | --- | --- | --- | --- | --- |
|  | | | | | | |
| **Compared groups** | | **difference** | **SE** | **Lower CI** | **Upper CI** | **p-value** |
| 0-5 | 6-10 | 900,500 | 556,7925 | -753,44 | 2554,439 | 0,5916 |
| 0-5 | 11-15 | 2678,821 | 402,9768 | 1481,79 | 3875,854 | <,0001* |
| 0-5 | 21-25 | 3911,875 | 454,6192 | 2561,44 | 5262,310 | <,0001* |
| 0-5 | 16-20 | 3585,519 | 408,5740 | 2371,86 | 4799,179 | <,0001* |
| 0-5 | 26-30 | 4605,583 | 491,0448 | 3146,95 | 6064,220 | <,0001* |
| 11-15 | 16-20 | 906,698 | 350,2063 | -133,58 | 1946,977 | 0,1202 |
| 11-15 | 21-25 | 1233,054 | 402,9768 | 36,02 | 2430,087 | 0,0400* |
| 11-15 | 26-30 | 1926,762 | 443,6629 | 608,87 | 3244,652 | 0,0010* |
| 16-20 | 21-25 | 326,356 | 408,5740 | -887,30 | 1540,015 | 0,9663 |
| 16-20 | 26-30 | 1020,064 | 448,7529 | -312,95 | 2353,074 | 0,2255 |
| 21-25 | 26-30 | 693,708 | 491,0448 | -764,93 | 2152,345 | 0,7192 |
| 6-10 | 11-15 | 1778,321 | 515,4897 | 247,07 | 3309,571 | 0,0143* |
| 6-10 | 16-20 | 2685,019 | 519,8769 | 1140,74 | 4229,301 | <,0001* |
| 6-10 | 21-25 | 3011,375 | 556,7925 | 1357,44 | 4665,314 | <,0001* |
| 6-10 | 26-30 | 3705,083 | 586,9108 | 1961,68 | 5448,488 | <,0001* |
| Shown is the post-hoc analysis with Tukey HSD for group comparison with significance level. The first two rows show the compared groups pairs. **SE**: standard error; **CI**: confidence interval | | | | | | |
