# Supplementary material for: From infancy to adulthood—Developmental changes in pulmonary quantitative computed tomography parameters
Source: PLoS One. 2020 May 29;15(5):e0233622. doi: 10.1371/journal.pone.0233622 (PMC7259551; doi:10.1371/journal.pone.0233622)
Supplement: S6 Table — (DOCX) [file pone.0233622.s007.docx]

| Table S6: comparison of age groups regarding - MLD Group 1 (non-contrast-enhanced) | | | | | | |
| --- | --- | --- | --- | --- | --- | --- |
|  | | | | | | |
| **Compared groups** | | **difference** | **SE** | **Lower CI** | **Upper CI** | **p-value** |
| 0-5 | 6-10 | 155,6250 | 23,03388 | 87,203 | 224,0466 | <,0001* |
| 0-5 | 26-30 | 227,1250 | 20,31397 | 166,783 | 287,4672 | <,0001* |
| 0-5 | 21-25 | 231,1250 | 18,80708 | 175,259 | 286,9910 | <,0001* |
| 0-5 | 11-15 | 220,1964 | 16,67070 | 170,677 | 269,7163 | <,0001* |
| 0-5 | 16-20 | 225,6250 | 16,90225 | 175,417 | 275,8327 | <,0001* |
| 11-15 | 16-20 | 5,4286 | 14,48764 | -37,607 | 48,4638 | 0,9990 |
| 11-15 | 26-30 | 6,9286 | 18,35383 | -47,591 | 61,4482 | 0,9989 |
| 11-15 | 21-25 | 10,9286 | 16,67070 | -38,591 | 60,4485 | 0,9859 |
| 16-20 | 26-30 | 1,5000 | 18,56440 | -53,645 | 56,6451 | 1,0000 |
| 16-20 | 21-25 | 5,5000 | 16,90225 | -44,708 | 55,7077 | 0,9995 |
| 26-30 | 21-25 | 4,0000 | 20,31397 | -56,342 | 64,3422 | 1,0000 |
| 6-10 | 26-30 | 71,5000 | 24,27984 | -0,623 | 143,6227 | 0,0532 |
| 6-10 | 11-15 | 64,5714 | 21,32523 | 1,225 | 127,9175 | 0,0434* |
| 6-10 | 16-20 | 70,0000 | 21,50672 | 6,115 | 133,8852 | 0,0242* |
| 6-10 | 21-25 | 75,5000 | 23,03388 | 7,078 | 143,9216 | 0,0228* |
| Shown is the post-hoc analysis with Tukey HSD for group comparison with significance level. The first two rows show the compared groups pairs. **MLD**: mean lung density; **SE**: standard error; **CI**: confidence interval | | | | | | |
