# Supplementary material for: From infancy to adulthood—Developmental changes in pulmonary quantitative computed tomography parameters
Source: PLoS One. 2020 May 29;15(5):e0233622. doi: 10.1371/journal.pone.0233622 (PMC7259551; doi:10.1371/journal.pone.0233622)
Supplement: S7 Table — (DOCX) [file pone.0233622.s008.docx]

| Table S7: comparison of age groups regarding FWHM - Group 1 (non-contrast-enhanced) | | | | | | |
| --- | --- | --- | --- | --- | --- | --- |
|  | | | | | | |
| **Compared groups** | | **difference** | **SE** | **Lower CI** | **Upper CI** | **p-value** |
| 0-5 | 6-10 | 80,1250 | 16,13717 | 32,1899 | 128,0601 | 0,0001* |
| 0-5 | 26-30 | 106,6250 | 14,23164 | 64,3502 | 148,8998 | <,0001* |
| 0-5 | 16-20 | 99,0865 | 11,84144 | 63,9118 | 134,2613 | <,0001* |
| 0-5 | 11-15 | 97,7679 | 11,67922 | 63,0750 | 132,4607 | <,0001* |
| 0-5 | 21-25 | 121,1250 | 13,17594 | 81,9862 | 160,2638 | <,0001* |
| 11-15 | 16-20 | 1,3187 | 10,14981 | -28,8311 | 31,4684 | 1,0000 |
| 11-15 | 26-30 | 8,8571 | 12,85840 | -29,3384 | 47,0527 | 0,9823 |
| 11-15 | 21-25 | 23,3571 | 11,67922 | -11,3357 | 58,0500 | 0,3578 |
| 16-20 | 26-30 | 7,5385 | 13,00592 | -31,0953 | 46,1722 | 0,9919 |
| 16-20 | 21-25 | 22,0385 | 11,84144 | -13,1363 | 57,2132 | 0,4381 |
| 26-30 | 21-25 | 14,5000 | 14,23164 | -27,7748 | 56,7748 | 0,9093 |
| 6-10 | 11-15 | 17,6429 | 14,94011 | -26,7364 | 62,0221 | 0,8436 |
| 6-10 | 16-20 | 18,9615 | 15,06727 | -25,7954 | 63,7185 | 0,8055 |
| 6-10 | 26-30 | 26,5000 | 17,01007 | -24,0280 | 77,0280 | 0,6295 |
| 6-10 | 21-25 | 41,0000 | 16,13717 | -6,9351 | 88,9351 | 0,1332 |
| Shown is the post-hoc analysis with Tukey HSD for group comparison with significance level. The first two rows show the compared groups pairs. **FWHM**: full width at half maximum; **SE**: standard error; **CI**: confidence interval | | | | | | |
