# Supplementary material for: From infancy to adulthood—Developmental changes in pulmonary quantitative computed tomography parameters
Source: PLoS One. 2020 May 29;15(5):e0233622. doi: 10.1371/journal.pone.0233622 (PMC7259551; doi:10.1371/journal.pone.0233622)
Supplement: S8 Table — (DOCX) [file pone.0233622.s009.docx]

| Table S8: comparison of age groups regarding LAV - Group 1 (non-contrast-enhanced) | | | | | | |
| --- | --- | --- | --- | --- | --- | --- |
|  | | | | | | |
| **Compared groups** | | **difference** | **SE** | **Lower CI** | **Upper CI** | **p-value** |
| 0-5 | 26-30 | 1,929167 | 0,7598356 | -0,32791 | 4,186240 | 0,1337 |
| 0-5 | 16-20 | 1,793269 | 0,6322214 | -0,08473 | 3,671268 | 0,0690 |
| 0-5 | 11-15 | 1,233929 | 0,6235604 | -0,61834 | 3,086200 | 0,3695 |
| 0-5 | 21-25 | 0,762500 | 0,7034711 | -1,32714 | 2,852144 | 0,8853 |
| 0-5 | 6-10 | 0,287500 | 0,8615726 | -2,27178 | 2,846781 | 0,9994 |
| 11-15 | 26-30 | 0,695238 | 0,6865175 | -1,34405 | 2,734522 | 0,9114 |
| 11-15 | 16-20 | 0,559341 | 0,5419041 | -1,05037 | 2,169054 | 0,9047 |
| 16-20 | 26-30 | 0,135897 | 0,6943936 | -1,92678 | 2,198577 | 1,0000 |
| 21-25 | 26-30 | 1,166667 | 0,7598356 | -1,09041 | 3,423740 | 0,6437 |
| 21-25 | 16-20 | 1,030769 | 0,6322214 | -0,84723 | 2,908768 | 0,5832 |
| 21-25 | 11-15 | 0,471429 | 0,6235604 | -1,38084 | 2,323700 | 0,9734 |
| 6-10 | 26-30 | 1,641667 | 0,9081772 | -1,05605 | 4,339385 | 0,4709 |
| 6-10 | 16-20 | 1,505769 | 0,8044499 | -0,88383 | 3,895369 | 0,4317 |
| 6-10 | 11-15 | 0,946429 | 0,7976612 | -1,42300 | 3,315862 | 0,8410 |
| 6-10 | 21-25 | 0,475000 | 0,8615726 | -2,08428 | 3,034281 | 0,9936 |
| Shown is the post-hoc analysis with Tukey HSD for group comparison with significance level. The first two rows show the compared groups pairs. **LAV**: low attenuated volume; **SE**: standard error; **CI**: confidence interval | | | | | | |
