# Supplementary material for: From infancy to adulthood—Developmental changes in pulmonary quantitative computed tomography parameters
Source: PLoS One. 2020 May 29;15(5):e0233622. doi: 10.1371/journal.pone.0233622 (PMC7259551; doi:10.1371/journal.pone.0233622)
Supplement: S9 Table — (DOCX) [file pone.0233622.s010.docx]

| Table S9: sex-demandant comparison of all parameters - Group 1 (non-contrast-enhanced) | | | | | |
| --- | --- | --- | --- | --- | --- |
|  | | | | | |
|  | | **♀** | **♂** | **Difference** | **p-value** |
| 0-5 | Volume | 530 ± 124 | 413 ± 110 | 118 | 0.2226 |
|  | MLD | -615 ± 46 | -555 ± 52 | 60 | 0.1762 |
|  | FWHM | 178 ± 43 | 226 ± 18 | 48 | 0.073 |
|  | LAV | 0 | 0.1 ± 0.17 | 0.1 | 0.4226 |
| 6-10 | Volume | 1524 ± 424 | 1250 ± 499 | 274 | 0.6146 |
|  | MLD | -792 ± 12 | -704 ± 94 | 87 | 0.4129 |
|  | FWHM | 101 ± 0.7 | 131 ± 40 | 30 | 0.4836 |
|  | LAV | 0.55 ± 0.21 | 0.1 ± 0.14 | 0.5 | 0.1481 |
| 11-15 | Volume | 3329 ± 909 | 3042 ± 670 | 287 | 0.5311 |
|  | MLD | -817 ± 23 | -809 ± 30 | 8 | 0.5844 |
|  | FWHM | 92 ± 15 | 102 ± 24 | 10 | 0.3788 |
|  | LAV | 1.5 ± 0.9 | 1.1 ± 0.9 | 0.3 | 0.4959 |
| 16-20 | Volume | 3173 ± 198 | 4843 ± 1366 | 1670 | 0.0175* |
|  | MLD | -809 ± 15 | -826 ± 30 | 17 | 0.2224 |
|  | FWHM | 104 ± 33 | 90 ± 13 | 13 | 0.394 |
|  | LAV | 1 ±0.8 | 2.5 ± 2.9 | 1.5 | 0.2196 |
| 21-25 | Volume | 4081 ± 268 | 4716 ± 864 | 635 | 0.241 |
|  | MLD | -818 ± 20 | -829 ± 28 | 12 | 0.5291 |
|  | FWHM | 72 ± 4 | 78 ± 29 | 6 | 0.7064 |
|  | LAV | 0.28 ± 0.4 | 1.3 ± 1.2 | 1 | 0.1678 |
| 26-30 | Volume | 4609 ± 4 | 5333 ± 1452 | 724 | 0.3921 |
|  | MLD | -828 ± 0 | -815 ± 53 | 13 | 0.6643 |
|  | FWHM | 81 ± 0 | 93 ± 27 | 12 | 0.4322 |
|  | LAV | 1.3 ± 0.6 | 2.3 ± 2 | 1 | 0.3943 |
| Shown is the students t-test for group-specific sex comparison of quantitative parameters **LAV**: low attenuated volume; **SE**: standard error; **CI**: confidence interval | | | | | |
