# Supplementary material for: From infancy to adulthood—Developmental changes in pulmonary quantitative computed tomography parameters
Source: PLoS One. 2020 May 29;15(5):e0233622. doi: 10.1371/journal.pone.0233622 (PMC7259551; doi:10.1371/journal.pone.0233622)
Supplement: S10 Table — (DOCX) [file pone.0233622.s011.docx]

| Table S10: sex-demandant comparison of all parameters - Group 2 (contrast-enhanced) | | | | | |
| --- | --- | --- | --- | --- | --- |
|  | | | | | |
|  | | **♀** | **♂** | **Difference** | **p-value** |
| 0-5 | Volume | 471 ± 338 | 394 ± 207 | 77 | 0.6666 |
|  | MLD | -500 ± 82 | -451 ± 66 | 49 | 0.333 |
|  | FWHM | 228 ± 60 | 250 ± 32 | 22 | 0.47 |
|  | LAV | 0.02 | 0 | 0.02 | 0.3632 |
| 6-10 | Volume | 715 ± 187 | 1659 ± 507 | 943 | 0.0267* |
|  | MLD | -488 ± 30 | -662 ± 118 | 174 | 0.0545 |
|  | FWHM | 234 ± 26 | 182 ± 131 | 52 | 0.4937 |
|  | LAV | 0 | 0.18 ± 0.2 | 0.18 | 0.1881 |
| 11-15 | Volume | 1996 ± 468 | 2384 ± 1220 | 388 | 0.4654 |
|  | MLD | -677 ± 19 | -705 ± 95 | 28 | 0.4471 |
|  | FWHM | 151 ± 32 | 134 ± 57 | 17 | 0.5442 |
|  | LAV | 0.1 ± 0.17 | 0.1 ± 0.24 | 0.013 | 0.9278 |
| 16-20 | Volume | 3715 ± 367 | 3651 ± 1112 | 63 | 0.8688 |
|  | MLD | -787 ± 34 | -741 ± 42 | 45 | 0.1262 |
|  | FWHM | 97 ± 17 | 111 ± 20 | 14 | 0.2712 |
|  | LAV | 0.67 ± 0.8 | 0.23 ± 0.42 | 0.44 | 0.4624 |
| 21-25 | Volume | 2992 ± 952 | 4793 ± 777 | 1801 | <.0001* |
|  | MLD | -729 ± 60 | -777 ± 31 | 48 | 0.0328* |
|  | FWHM | 128 ± 64 | 105 ± 15 | 23 | 0.2734 |
|  | LAV | 0.1 ± 1.3 | 0.7 ± 0.6 | 0.58 | 0.0083* |
| 26-30 | Volume | 3556 ± 692 | 5151 ± 1285 | 1595 | 0.0002 |
|  | MLD | -754 ± 50 | -780 ± 58 | 26 | 0.2281 |
|  | FWHM | 111 ± 27 | 102 ± 27 | 7.8 | 0.484 |
|  | LAV | 1.35 ± 3.3 | 1.1 ± 1.1 | 0.25 | 0.8287 |
| Shown is the students t-test for group-specific sex comparison of quantitative parameters **LAV**: low attenuated volume; **SE**: standard error; **CI**: confidence interval | | | | | |
